# Catecholamine-Induced Myocarditis in Undetected Pheochromocytoma

**DOI:** 10.7759/cureus.85720

**Published:** 2025-06-10

**Authors:** Narmeen Hassan, Jing W Goh, Usman Hassan

**Affiliations:** 1 Internal Medicine, Countess of Chester Hospital, Chester, GBR; 2 Respiratory Medicine, Countess of Chester Hospital, Chester, GBR; 3 Geriatric Medicine, Countess of Chester Hospital, Chester, GBR

**Keywords:** adrenal pheochromocytoma, myocarditis, pheochromocytoma crisis, severe hypertension, unexplained shock

## Abstract

Pheochromocytoma is a neuroendocrine tumor of the adrenal gland. It can cause a range of symptoms, but the main ones are palpitations, pallor, headaches, and sweating. Due to its rarity and episodic character, it is frequently misdiagnosed in the initial instance.

We report the case of a young woman in her twenties who was misdiagnosed with hypotension, headache, cough, and palpitations, and an echocardiography revealed reduced cardiac function. She was initially diagnosed with Takotsubo cardiomyopathy and then with myocarditis based on clinical and radiological findings, and she was treated for cardiogenic shock. She continued to have a headache, which was diagnosed as a cardiac cephalalgia. Despite receiving topiramate and analgesia, she continued to have headaches and hypotension. She was found to have periodic episodes of hypertension, prompting a pheochromocytoma screening. She had a successful adrenalectomy and recovered well. This case report emphasizes the significance of having a high degree of suspicion for pheochromocytoma in individuals presenting with unexplained cardiac dysfunction.

## Introduction

Pheochromocytoma and paraganglioma (PPGL) are uncommon neuroendocrine tumors that originate from chromaffin cells. They produce an excessive amount of catecholamines, especially norepinephrine and epinephrine. These catecholamines may result in a number of symptoms once they enter the circulatory system, including anxiety, pallor, sweating, palpitations, hypertension, and recurrent headaches [1]. Excess catecholamines in the blood have detrimental effects on the heart and blood vessels, leading to complications, including myocarditis and cardiomyopathy. 

Early diagnosis and treatment can lead to reversibility of the cardiomyopathy; however, due to its wide range of symptoms, diagnosis is often delayed, which can be detrimental [2]. Medical management of pheochromocytoma includes treatment with alpha-adrenergic blockers, perhaps along with beta-adrenergic blockers to avoid symptoms related to beta-adrenoceptor stimulation, such as tachycardia. Definitive treatment remains surgical excision of the tumor [3].

## Case presentation

A 21-year-old woman with no known medical history went to the emergency department (ED) of a local district general hospital with a worsening intermittent headache over the past four months, followed by vomiting and a productive cough. Prior to hospitalization, her primary care physician diagnosed her with a migraine and prescribed sumatriptan, which she did not respond to. She was admitted with hypotension, and her initial blood tests revealed elevated inflammatory markers and raised troponin (Table 1). She was prescribed broad-spectrum antibiotics for an infection of unknown origin, possibly leading to myocarditis. Due to the worsening of her clinical condition, including severe hypotension, she was transferred to the ICU for inotropic support. 

**Table 1:** Patient's blood report on initial presentation CRP: C-reactive protein, eGFR: Estimated glomerular filtration rate

Parameter	Value	Reference range
WBC	31.6 x 10^9^/L	4.0-11.0 x 10^9^/L
Platelets	703 x 10^9^/L	120-400 x 10^9^/L
Hemoglobin	190 g/L	115-160 g/L
Neutrophils	25.00 x 10^9^/L	2.0-6.0 x 10^9^/L
Lymphocytes	5.10 x 0^9^/L	1.0-3.50 x 0^9^/L
D-dimer	8970	0-500
Sodium	140 mmol/L	133-144 mmol/L
Potassium	4.6 mmol/L	3.5-5.0 mmol/L
Urea	6.1 mmol/L	2.0-6.5 mmol/L
Creatinine	118 umol/L	50-120 umol/L
Lactate	3.86 mmol/L	0.63-2.44 mmol/L
CRP	8 mg/L	0-10 mg/L
Troponin I	394.9 ng/L	0.0- 11.6 ng/L
eGFR	57 ml/min	60-250 ml/min
Procalcitonin	2.97 ug/L	<= 0.50 ug/L

An echocardiography (echo) was performed, which revealed markedly decreased left and right ventricular function. In the ICU, the patient was treated conservatively with angiotensin-converting enzyme (ACE) inhibitors, a beta-blocker, a mineralocorticoid receptor antagonist, and a course of intravenous steroids for shock. A repeat echo 48 hours later showed remarkable recovery, including normal left ventricular (LV) function and a slightly dilated right ventricle (RV) (Video 1). Her inflammatory markers, although still raised, started to show improvement as well. She was later transferred from the ICU to the cardiology ward for further care, with a preliminary diagnosis of Takotsubo cardiomyopathy, possibly caused by recent stress at home.

**Video 1:** Echocardiography showing severe global impairment and then its recovery

The patient's headaches persisted; therefore, a consultation with a neurology specialist was sought. It was thought that her headaches could be caused by cardiac cephalgia. She was advised to start topiramate and have a urine test for metanephrines. However, no pheochromocytoma screening tests were performed because the patient did not have hypertension. 

A cardiac MRI was performed to determine the etiology of her cardiac dysfunction. It revealed ill-defined areas of increased T2 myocardial time, more prominent in the apical wall (anterior, septal, and anterolateral), as well as the entire mid-wall, septal, and lateral basal regions. The maximum T2 time measured 60 msec (basal septum). The same areas showed increased native T1 time (basal septum: 1113 msec). There was no delayed enhancement, ischemic scar, or infiltration noted. These findings were suggestive of an inflammatory condition. The MRI details were reassuring, showing that her LV systolic function had recovered to normal. The findings were most consistent with acute myocarditis, although her viral screen was negative (Table 2), and she was subsequently discharged home.

**Table 2:** Result of viral screen CMV: Cytomegalovirus, EBV VCA: Epstein-Barr virus viral capsid antigen, EBNA: Epstein-Barr virus nuclear antigen, PCR: Polymerase chain reaction

Parameter	Value	Reference
CMV IgG antibody	Detected	Positive test indicates previous infection
CMV IgM antibody	Not detected	Positive test indicates recent infection
EBV VCA IgG EIA	Positive	Positive test indicates past EBV infection
EBV VCA IgM EIA	Negative	Positive test indicates recent primary EBV infection
EBNA IgG	Present	Positive test indicates past EBV infection
Hepatitis B surface antigen	Not detected	-
Hepatitis C antibody	Not detected	-
HIV 1+2 antibody	Not detected	-
HIV p24	Not detected	-
Herpes simplex virus 1	Not detected	-
Herpes simplex virus 2	Not detected	-
Adenovirus PCR	Negative	-
Parvovirus B19 IgG antibody	Detected	Positive test indicates past infection and immunity
Parvovirus B19 IgM antibody	Not detected	Positive test indicates current infection

**Figure 1:**
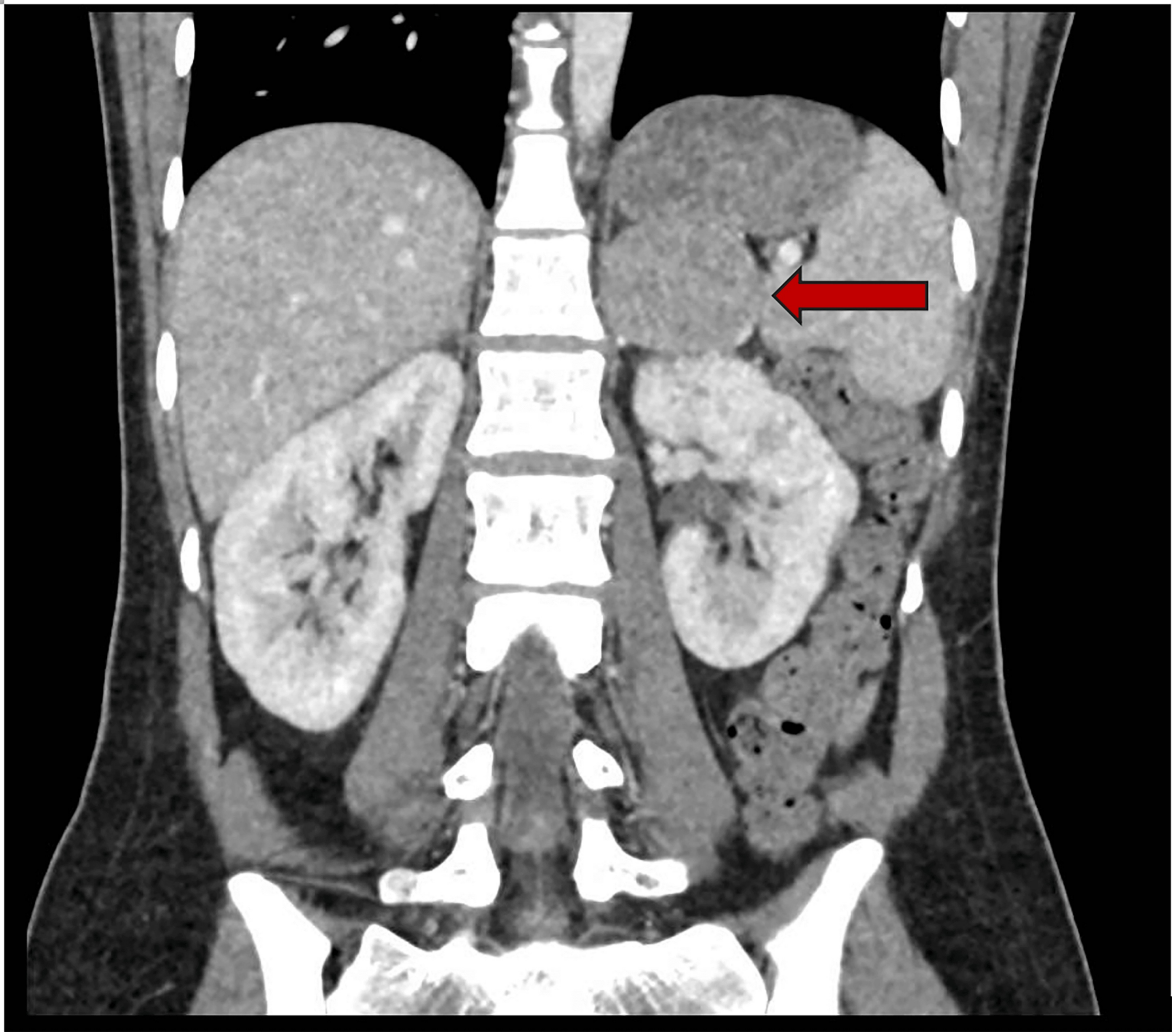
CT abdomen The red arrow points to the left adrenal mass (88 HU).

**Figure 2:**
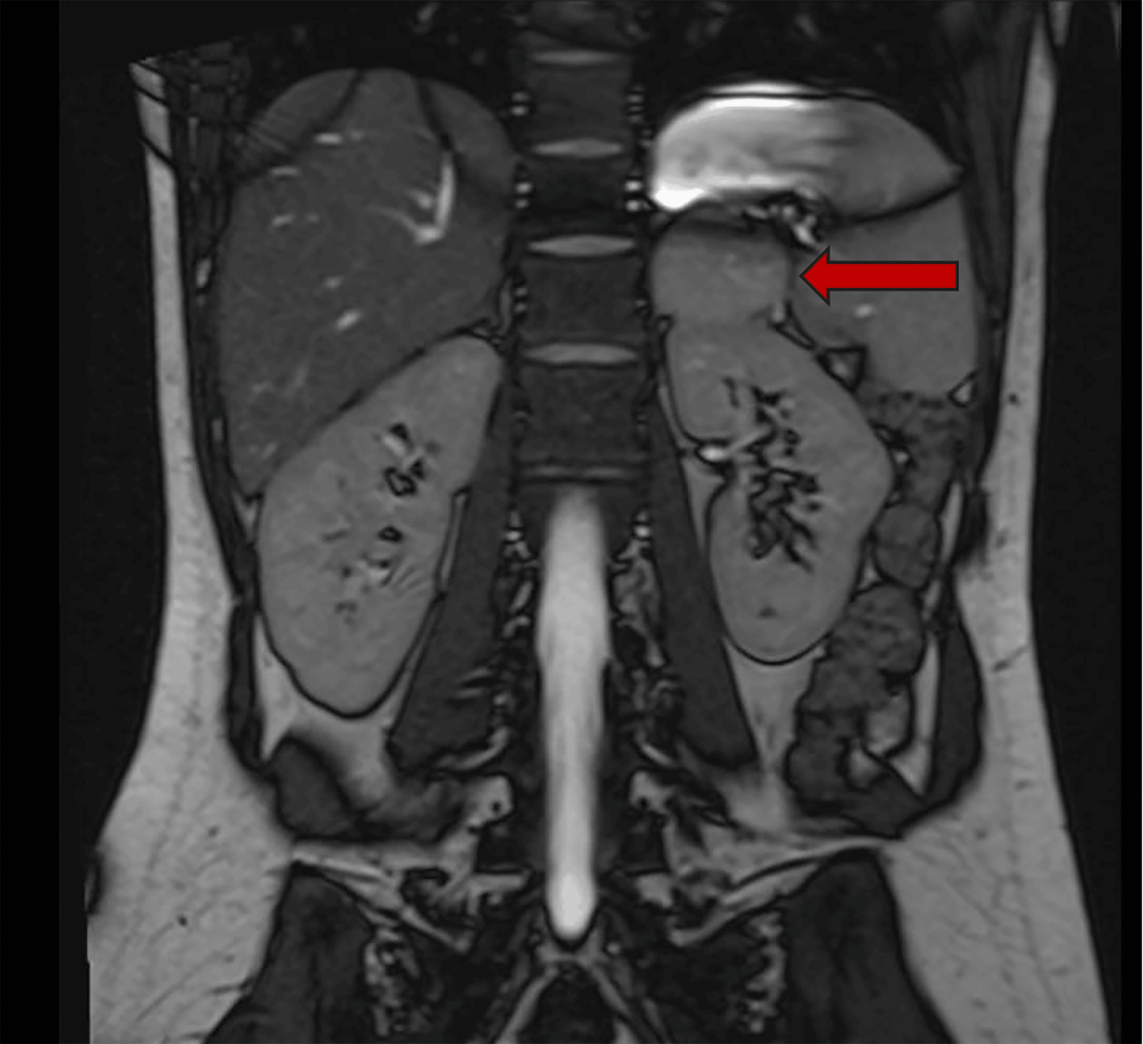
MRI of the adrenal gland The red arrow points to the left adrenal mass.

She returned to the ED within 48 hours with symptoms similar to her previous presentation, including headache, vomiting, palpitations, and low blood pressure. A bedside echo revealed good LV contractility. Her cardioprotective drugs were discontinued due to the echo findings and her persistently low blood pressure. Despite this, her inflammatory markers increased during the next three days, and she developed a fever, hypotension, and an acute kidney injury. She was treated conservatively with intravenous fluids and broad-spectrum antibiotics for an infection of unknown origin. As a result, her blood pressure improved. She developed high blood pressure intermittently, with a systolic BP of 200 mmHg. This prompted further assessment of pheochromocytoma, including a 24-hour urinary metanephrine, plasma metanephrine, and CT abdomen, which revealed a tumor on the left adrenal gland measuring 46 mm × 44 mm with homogeneous attenuation (88 HU) (Figure 1). She then underwent an MRI of her adrenals (Figure 2), which revealed an adrenal mass. However, this was classified as an uncertain scan, given that the adrenal mass differential included a lipid-poor functional adenoma, a pheochromocytoma, and an adrenal cortical carcinoma. 

**Figure 3:**
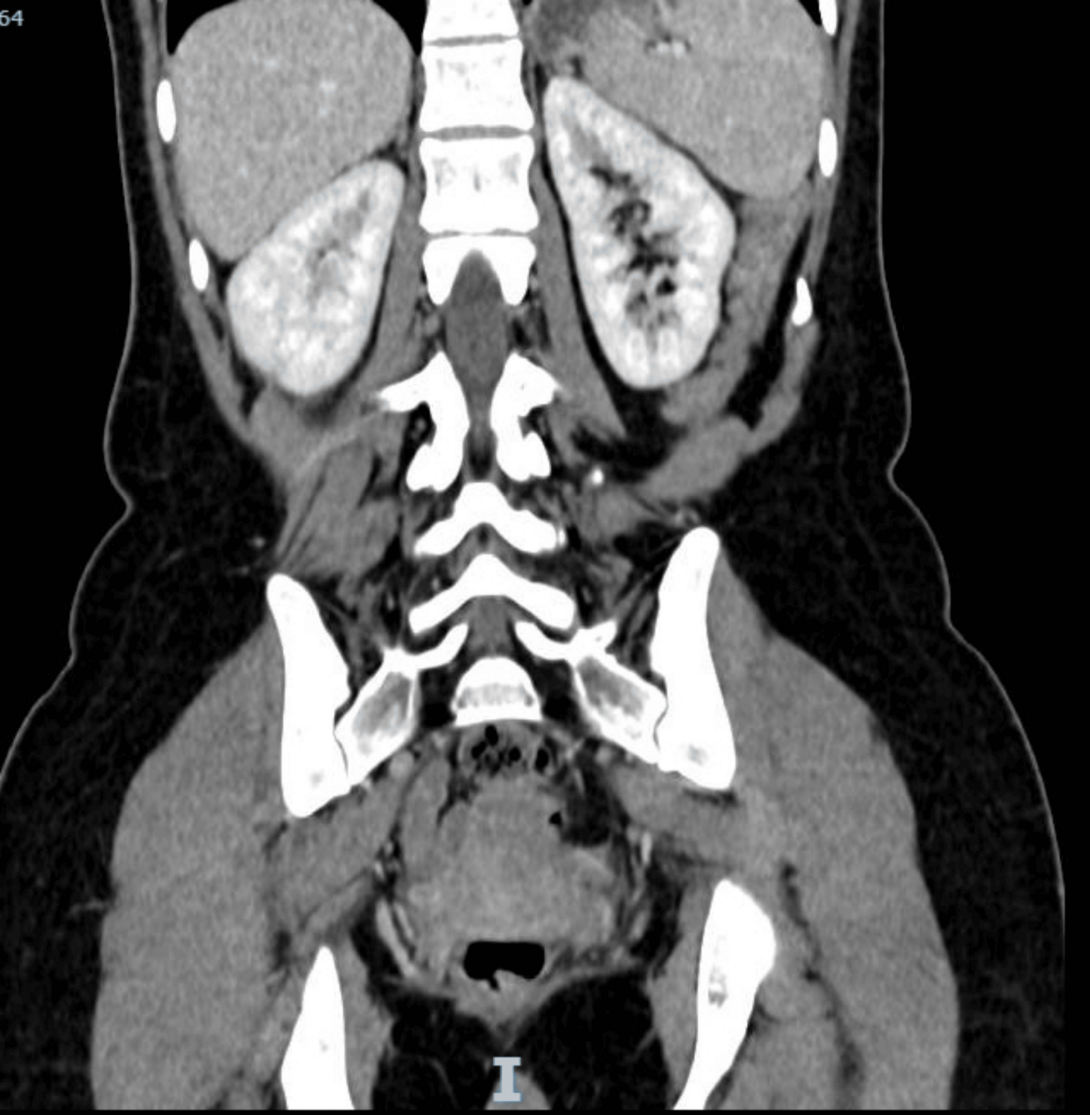
Postoperative CT scan confirming removal of adrenal tumor

Her urine and plasma metanephrine levels were elevated, indicating pheochromocytoma with adrenergic phenotype (Table 3). Following the diagnosis, she was evaluated by an endocrinologist, who prescribed an alpha blocker, which resulted in the normalization of her blood pressure. She was subsequently discharged from the hospital after starting beta blockers and being referred for an adrenalectomy. She underwent surgery and recovered well, with her headaches, palpitations, and shortness of breath resolved. A biopsy of the tumor confirmed the diagnosis of left adrenal pheochromocytoma pT2 pNx with retained expression of succinate dehydrogenase complex subunit B on immunohistochemistry. Following surgery, the endocrinologist will closely monitor plasma metadrenalines, conduct genetic testing for multiple endocrine neoplasia (MEN), and have a repeat CT abdomen performed at the three-month follow-up (Figure 3).

**Table 3:** The patient's urinary and plasma metanephrines test results indicating adrenergic biochemical phenotype

Parameter	Value	Reference upper limit
Plasma metanephrine	5.2 nm/L	0.5 nm/L
Plasma normetanephrine	9.11 nm/L	1.18 nm/L
Urine normetadrenaline	12.15	-
Urine 24-hour normetadrenaline	16.65 umol/day	3.30 umol/day
Urine metadrenaline	31.86	-
Urine 24-hour metadrenaline	43.65 umol/day	1.20 umol/day

## Discussion

Catecholamine-induced myocarditis has been widely reported in many studies as one of the clinical manifestations of pheochromocytoma. However, it remains a rare clinical presentation. Patients with pheochromocytoma develop catecholamine-related signs and symptoms. Excess catecholamine stimulation can produce severe vasoconstriction, coronary vasospasm, and myocardial ischemia, resulting in tissue damage and necrosis. Moreover, persistently elevated catecholamine levels might cause cardiac dysfunction by desensitizing cardiac β-adrenoceptors [4].

In this case, our patient was diagnosed with myocarditis based on clinical and radiological symptoms despite the lack of a specific cause. Nonetheless, her clinical symptoms were consistent with myocarditis. The eventual cause of myocarditis, pheochromocytoma, was subsequently identified because of her hypertension and uncontrollable headache, prompting further investigation. As a result, it is critical to underline the significance of maintaining a high level of suspicion for pheochromocytoma in individuals with severe cardiovascular symptoms. This is especially important when the clinical trajectory deviates from the usual course of myocarditis or demonstrates unresponsiveness to standard treatment [5].

The typical symptoms of PPGLs, namely headache, palpitations, and sweating, sometimes known as the pheochromocytoma triad, are widely described. However, these symptoms lack specificity because they can appear in a variety of other conditions, notably those involving elevated sympathetic nervous system activity. While the triad is 90% specific for identifying pheochromocytoma, it is seldom seen, appearing in 10% to 36.5% of cases [6]. Patients with pheochromocytoma may have labile blood pressure due to the episodic release of catecholamines [7]. Furthermore, this typical pheochromocytoma triad might appear in various illnesses, such as thyrotoxicosis, hypoglycemia, anxiety or panic attacks, hypercortisolism, hyperaldosteronism, and intracranial lesions, making the diagnosis more challenging [8]. Therefore, clinicians should maintain a high degree of suspicion for pheochromocytoma in patients with difficult-to-control hypertension, despite its occurrence in less than 1% of hypertensive patients [8].

The patient was diagnosed with acute myocarditis with migraine after first presenting with migraines and low blood pressure. Pheochromocytoma was not included in the differentials since it is frequently linked to high blood pressure instead of low blood pressure. In fact, pheochromocytoma patients may have either high or low blood pressure, along with additional symptoms such as fever, encephalopathy, and multiorgan failure. According to the literature, patients with pheochromocytoma may experience rapid and severe hemodynamic instability due to an excess of catecholamines, resulting in organ damage or dysfunction [9]. This is known as the pheochromocytoma crisis (PCC). It has been reported that PCC has a 15% mortality rate [10]. As a result, early diagnosis and treatment of pheochromocytoma are crucial in clinical practice.

Diagnosis of pheochromocytoma often involves combining clinical observations, biochemical tests, and imaging studies. For patients showing typical symptoms of pheochromocytoma, measuring plasma or urine metanephrines is essential to assist the diagnosis. The study has shown that urinary total metanephrines and catecholamines have a sensitivity rate of 90%, while fractionated plasma metanephrines demonstrate a sensitivity of 97% in the diagnosis of pheochromocytoma. Regarding specificity, urinary tests demonstrate a higher rate of 98% compared to the 85% specificity of fractionated plasma metanephrines [11]. Radiological investigations such as CT and MRI play a vital role in identifying both adrenal and extra-adrenal tumors. According to the study by Lumachi et al., the sensitivities for CT and MRI are 90% and 93%, respectively, with 93% specificity for both modalities. Nuclear medicine scans, such as the meta-iodobenzylguanidine (MIBG) scan, may be used to obtain a more thorough assessment of adrenal mass [12]. A definitive diagnosis is ultimately established by correlating biochemical findings with imaging results, often confirmed through histological analysis following surgical excision of the tumor.

In this case, the patient's symptoms of high blood pressure and headaches prompted the clinician to order an abdominal CT scan to check for pheochromocytoma while awaiting the results of urinary and plasma metanephrine tests. Although the MRI of the adrenal glands was inconclusive, starting treatment for pheochromocytoma based on clinical suspicion led to stabilization and significant improvement in the patient's condition. However, surgical removal remains the only definitive treatment for pheochromocytoma. Preoperative preparation with adrenergic blocking medications until the patient achieves normal blood pressure and is free from episodes has greatly reduced the previously high rates of surgical complications [13].

## Conclusions

Pheochromocytoma is a rare and potentially life-threatening neuroendocrine tumor originating from the adrenal medulla. It is characterized by excessive production of catecholamines, resulting in a wide variety of symptoms, including hypertension, tachycardia, headache, sweating, anxiety, and palpitations. Given the variability of the symptoms, it is often misdiagnosed. A high clinical suspicion is needed in individuals with atypical presentation. It can be diagnosed by biochemical testing, i.e., plasma and urinary metanephrines, and imaging such as CT or MRI of the adrenals and the MIBG scan in some cases. Treatment includes medical management with alpha blockers followed by beta blockers. The definitive treatment is surgical resection of the tumor. 
